# Analysis of Patients Diagnosed with Benign Paroxysmal Positional Vertigo and the Corresponding Incidence and Patterns of Electric Toothbrush Use

**DOI:** 10.7759/cureus.5697

**Published:** 2019-09-19

**Authors:** Navdeep R Sayal, Eric L Cox, Nicholas Foster, Matthew Globerson, Matthew Farrugia

**Affiliations:** 1 Otolaryngology, Beaumont Health, Farmington Hills Campus, Farmington Hills, USA; 2 Otolaryngology, Michigan State University College of Osteopathic Medicine, East Lansing, USA

**Keywords:** vertigo, bppv, recalcitrant

## Abstract

Objective

To investigate whether mechanical vibrational energy from using an electric toothbrush may cause an increase in the incidence of benign paroxysmal positional vertigo (BPPV) and prevent successful treatment of BPPV with canalith repositioning procedure.

Methods

This was a retrospective study conducted at an otolaryngology private practice. A survey of 111 patients who were diagnosed with BPPV in an otolaryngology practice between May 2012 and January 2017 was conducted using a questionnaire that included questions regarding demographics, inner ear pathology, treatment method, and use of an electric toothbrush. The results were recorded and compared using a chi-square test of analysis or Fisher’s exact test.

Results

Overall, 47 (42.3%) of the 111 BPPV patients used an electric toothbrush, whereas 64 of the 111 (57.6%) patients did not. Six (12.7%) of the 47 patients experienced dizziness with electric toothbrush use. Of the 47 patients using an electric toothbrush, 33 (70.2%) had a resolution of symptoms after Epley treatment, whereas 14 (23.4%) of 47 patients did not. Of the 64 patients who did not use an electric toothbrush, 15 (23.4%) did not have resolution after Epley treatment. Of the 47 patients using an electric toothbrush, 6 (12.8%) had a recurrence of BPPV diagnosed in the office, whereas 41 (82.2%) did not. Seven (10.9%) of those who did not use an electric toothbrush had a recurrence of BPPV, whereas 57 (89.1%) of the 64 patients did not. These results were not statistically significant (p = 0.77).

Conclusions

This study suggests that the mechanical vibrations from electric toothbrush use do not have an association with recurrent BPPV. The results align with some publications demonstrating that vibrations in the head and neck area from the use of an electric toothbrush can initiate dizziness; however, it does not appear that this modality of vibration is significant for inducing recurrent BPPV.

## Introduction

Benign paroxysmal positional vertigo (BPPV) is the most common type of vestibular disorder, with a lifetime prevalence estimated at 2.4% in the general population [1]. BPPV is typically idiopathic but can occur after head trauma or secondary to various disorders that damage the inner ear and detach otoliths from the utricle [2]. A possible explanation of this type of vertigo could be based on the detachment of fragments from the utricle’s otolithic membrane. The particles that detach from the utricle and accumulate in the semicircular canal may agglomerate and act like a piston on the endolymph, causing a shift in the cupula and therefore inducing vertigo and nystagmus [3]. BPPV can be diagnosed using provocation maneuvers such as the Dix-Hallpike and Pagnini-McClure tests [2]. Canalith repositioning procedures effectively treat BPPV, but despite the demonstrated efficacy of these maneuvers and the possible spontaneous resolution, symptoms may persist [3].

Researchers have reported that vibratory stimuli of the head and neck could induce nystagmus in patients with vestibular disorders [4]. Lackner and Graybiel were the first to observe vestibular side effects from the regional vibration of the skull that caused an illusion of motion in normal subjects [5]. Chang et al. demonstrated a correlation between dental procedures and BPPV. They demonstrated a correlation between dental procedures and BPPV, in which they hypothesized that the vibratory and percussive tools in dental therapy directly induce otolith redistribution. Furthermore, the energy of the vibratory or percussive impacts is transferred through the bone, thereby entering the labyrinths and loosening or dislodging otoliths. [6]. BPPV following mountain biking or after using a whole-body vibration training plate has also been reported, providing further evidence supporting the mechanical causes of BPPV [7]. Electric toothbrushes transmit mechanical vibrations to the skull in a similar manner as dental tools, but with less intense vibration. Chang et al. hypothesized that dental procedures may initially loosen otoconia, and dislodgement of otoliths may be delayed for days, weeks, or even months [6]. Their theory led to the speculation that even though electric toothbrush use does not provide the same vibratory energy to the skull as dental tools, the daily low-energy vibrations may provide enough energy to loosen otoliths over time, in a similar fashion as previously hypothesized from dental procedures.

The aim of this study was to investigate if the mechanical vibrational energy from electric toothbrush use may cause otoconia to become dislodged, thus increasing the incidence of BPPV. Specifically, we were interested in whether the use of an electric toothbrush prevents successful treatment of BPPV, with the canalith repositioning procedure acting as a mechanism for recalcitrant BPPV. We hypothesized that there was a relationship between electric toothbrush use and recalcitrant BPPV.

## Materials and methods

A chart review was conducted of all the patients diagnosed with BPPV in an otolaryngology private practice between May 2012 and January 2017. A positive diagnosis of BPPV was defined as the presence of nystagmus elicited in a Dix-Hallpike maneuver by a physician in the practice. We directly surveyed a total of 404 patients with BPPV from this group using a questionnaire that included questions regarding demographics, diagnosis of an inner ear disorder, diagnosis of BPPV, treatment method, improvement of BPPV, and use of an electric toothbrush. Of these patients, 111 responded to our survey. Patients who answered yes to the use of electric toothbrushes were asked the questions regarding the following: length of time using an electric toothbrush, frequency of electric toothbrush use, whether the patient experienced dizziness while using their electric toothbrush, and whether the patient used another vibration device (i.e. lawnmower, electric beard trimmer, etc.). The inclusion criteria included any patient older than 18 years who was seen at the otolaryngology clinic. The exclusion criteria excluded patients under the age of 18 years. The patients were surveyed through phone calls or direct questionnaires. The Institutional Review Board at Beaumont Hospital, Farmington Hills, reviewed and approved all aspects of the study, including the content of the survey, in accordance with institutional, federal, and international guidelines.

Categorical data were summarized as counts and percentages and were compared using the chi-square test for association or Fisher’s exact test. Throughout this study, a p-value of ≤0.05 (two-tailed) was considered statistically significant. Following initial data entry using Microsoft Excel, analyses were performed using Minitab Statistical Software (State College, PA) or VassarStats online calculator (Richard Lowry, http://vassarstats.net/tab2x2.html) [8].

## Results

Overall, 47 (42.3%) of 111 BPPV patients used an electric toothbrush, whereas 64 (57.6%) did not. The female-to-male ratio of electric toothbrush users was 2.6:1 (34 females and 13 males, respectively). The female-to-male ratio of users who did not use an electric toothbrush was 1.6:1 (40 females and 24 males, respectively). The difference in these ratios was not statistically significant (p = 0.27).

In addition, 41 (87.2%) of the 47 patients who used an electric toothbrush and 57 (89.1%) of the 64 patients who did not use an electric toothbrush reported no other inner ear disorder; 45 (95.7%) of 47 electric toothbrush users and 61 (95.3%) of 64 nonelectric toothbrush users did not use another vibrational device. Of the electric toothbrush users, the average time patients had used one was approximately 8.1 years. The average length of time spent brushing their teeth per use was 2.1 minutes. Of the 47 electric toothbrush users, 12 (25.5%) brushed once daily, 33 (70.2%) brushed twice daily, and two (4.3%) brushed three times daily with an electric toothbrush (Table 1).

**Table 1:** Electric Toothbrush Use Information

	Electric toothbrush users (N = 47)
Estimated time of use (months)	98 months (8.1 years)
Time per use (minutes)	2.1
Times per day used	
1	25.5%
2	70.2%
3	4.3%
Dizziness while using an electric toothbrush	
Yes	12.7%
No	82.2%

Of the 47 patients, 6 (12.8%) became dizzy with electric toothbrush use, whereas 41 (82.2%) did not. In addition, 41 (87.2%) patients did not use any medication for BPPV, 4 (8.5%) used meclizine, 1 (2.1%) used metoprolol, and 1 (2.1%) used betahistine.

Of the 47 patients who used an electric toothbrush, 33 (70.2%) had a resolution of symptoms after Epley treatment. Of the 64 patients who did not use an electric toothbrush, 49 (76.6%) had resolution with Epley treatment, whereas 15 (23.4%) did not. Of the 47 electric toothbrush users, 6 (12.8%) had a recurrence of BPPV diagnosed in the office, whereas 41 patients (82.2%) did not. Among those who did not use an electric toothbrush, 7 (10.9%) of 64 patients had a recurrence of BPPV, whereas 57 (89.1%) did not. These results were not statistically significant (p = 0.77). For the electric toothbrush users with recurrence, three (50%) of six patients were treated two times before resolution, one (16.7%) was treated three times before resolution, one (16.7%) was treated four times before the resolution of symptoms, and one (16.7%) was treated eight times. Of the seven patients not using electric toothbrushes who had a recurrence of symptoms, one (14.3%) was treated twice before the resolution of symptoms and three (42.9%) were treated three times before resolution, with the other three (42.9%) either requiring surgery to correct the symptoms or experiencing symptoms at the time of the survey (Table 2).

**Table 2:** General Information in Relation to Electric Toothbrush Use Note: the differences between electric toothbrush users and non-users were not statistically significant (p > 0.05).

	Electric toothbrush users (N = 47)	Non-electric toothbrush users (N = 64)
Gender		
Male	27.7%	37.5%
Female	72.3%	62.5%
Another inner ear pathology/disorder		
No	87.2%	89.1%
Tinnitus	8.5%	1.6%
Otosclerosis	2.1%	9.4%
Eustachian tube dysfunction	2.1%	9.4%
Another device used		
No	95.7%	95.3%
2	2.1%	4.7%
3	2.1%	0%
Medications used		
None	87.2%	84.4%
Meclizine	8.5%	15.6%
Metoprolol	2.1%	0%
Betahistine	2.1%	0%
Symptoms resolve after the first Epley treatment		
Yes	70.2%	76.6%
No	29.8%	23.4%
Recurrence of BPPV		
Yes	12.8%	10.9%
No	82.2%	89.1%
If recurrence, how many more times treated		
2	50%	14.4%
3	16.7%	42.9%
4	16.7%	0%
>4	16.7%	0%

## Discussion

Although the possible improvement in oral hygiene may justify electric toothbrush use, the vibrations the instrument produces in patients with a history of vestibular diseases may exacerbate vertiginous symptoms. Regional vibration to the skull that triggered vertiginous symptoms and an illusion of motion was first demonstrated by Lackner and Graybiel [5]. Ohki et al. demonstrated vibration’s role in producing nystagmus in patients with unilateral vestibular dysfunction [9]. Chang et al. showed that dental procedures carried a 1.77-fold increase in the odds of patients having BPPV compared with patients who did not undergo any dental procedures [6].

Two strongly associated risk factors categories that potentially induce the dislodging of the otoliths are vascular and mechanical [6]. One example of a vascular risk factor noted in the literature is the association between migraines and BPPV [10-11], with its pathophysiology hypothesized to be vascular dilation and extravasation of fluid or vasospasm. Chronic cardiovascular diseases such as hypertension, hyperlipidemia, diabetes, and atherosclerosis are also vascular risk factors and may predispose individuals to BPPV [12-13]. Mechanical risk factors include a wide spectrum of types of head movements, including BPPV secondary to mild head trauma to subtle vibrations that can induce nystagmus and vertigo [14]. Other investigations of BPPV in patients without a history of head trauma include one by Vibert et al. that showed an immediate onset of BPPV after intense mountain biking in four patients, which was attributed to the forceful accelerations/decelerations that occurred during jumps and turns throughout a mountain biking course [7]. One case report documented the triggering of BPPV in a 44-year-old female for a duration of one week after she used a whole-body vibration plate at her fitness center [15].

The mainstay treatment method for BPPV is the Epley maneuver, which uses gravity to reposition the otoliths outside the affected semicircular canals and into the utricle, where it can no longer trigger the hair cells and create symptomatic vertigo [1]. Adding mastoid vibration during the head positioning maneuvers is also thought to improve the outcomes of initial treatments by assisting in the mobilization of otoliths during maneuvers, although a study by Macias et al. found contradictory data. The researchers noted that there was a relapse rate of 30.5% in patients treated with mastoid vibration during the initial Epley manuever, with a trend that demonstrated greater propensity for relapse as follow-up time increased [16]. Antihistamine medication and benzodiazepines can assist in alleviating symptoms but do not treat the underlying etiology of BPPV. Surgical options also exist but are invasive and reserved for severe cases that are refractory to head positioning maneuvers, and they carry a risk of complications postoperatively [17].

A study by Teggi et al. investigated recurrent BPPV in patients after an initial repositioning head maneuver. They found that 31.1% of patients reported recurrent BPPV on the second day after the initial treatment and that a correlation existed between the duration of BPPV and the occurrence of recurrent BPPV [18]. Researchers defined residual dizziness as an imbalance without positional vertigo and concluded that multiple factors might influence residual dizziness, including the persistence of otoliths, undiagnosed vestibular pathology, and a physiological adaptation to a previously untreated vestibular disorder [1]. Seok et al. followed 49 patients and found that more than 60% of the patients complained of residual dizziness after repositioning treatment and that an earlier initial intervention with head maneuvers was associated with a decreased incidence of residual dizziness [19]. 

Our data showed that 29 (26.1%) participants stated that their BPPV symptoms did not resolve after the first head positioning maneuver treatment. Of these participants, 14 were electric toothbrush users. There was no statistical significance between the electric toothbrush use group and the non-electric toothbrush use group associated with symptom resolution after one head positioning maneuver treatment. In both the groups, approximately one-quarter of their respective participants stated that their symptoms did not resolve. Our data also showed that a higher percentage of patients experienced recurrence after initial head positioning maneuver treatment in the electric toothbrush user group (six users [12.8%]) compared with the non-electric toothbrush user group (seven users [10.9%]), but this difference was not statistically significant (p = 0.77).

Additionally, of the 47 study participants who use electric toothbrushes, 6 (12.8%) reported dizziness when using their electric toothbrushes. The data collected suggest that the vibrations produced during electric toothbrush use have a minimal effect on unresolved BPPV after an initial head positioning maneuver treatment, as well as recurrence of BPPV when comparing electric toothbrush users with non-electric toothbrush users. Multiple confounding factors such as medications, use of other vibratory devices, and associated inner ear disorders were similar across each group and do not seem to influence the results obtained when comparing the two groups.

One limitation of the study is that the specific dates of initial BPPV diagnosis, initiation of electric toothbrush use, and the use of electric toothbrushes relative to the initial head positioning maneuver treatment were not established. More specific timelines included in a subsequent study may prove beneficial in determining a possible link between electric toothbrush use and recurrent BPPV. Another limitation to the study is the recall bias of the patients pertaining to their electric toothbrush use. It would have been important to ellicit if the patients experiencing symptomatic recurrence had been using electric toothbrushes prior to initial Epley treatment. Further investigations into the etiology of BPPV recurrence and the association with multiple factors, including patient compliance with post-Epley instructions, noise exposure after initial treatment, and blood pressure regulation following Epley, would be warranted.

Clinically, the findings of this study suggest that electric toothbrush use does not increase the risk of persistent BPPV symptoms with appropriate therapy. The findings are limited to the population surveyed, and therefore physicians should be cognizant of geographic disparities that may have affected the data, although all participants were randomized and there were no statistically significant characteristic differences among the population surveyed. Of course, with the limitations of this study previously stated, it is imperative to discuss with the patient that the recurrence of BPPV symptoms may return in light of vibrational device use. Post-Epley instructions should always be given to the patient in order to increase the likelihood of symptom resolution, and with the data found in this study, patients may be informed that electric toothbrush use can safely be continued. 

## Conclusions

Based on our data, this study suggests that the mechanical vibrations from electric toothbrushes do not have an association with recurrent BPPV. A small subset of BPPV patients in the study did experience dizziness with electric toothbrush use. The results align with some publications that demonstrate that dizziness can be initiated with vibrations in the head and neck area; however, this study suggests that an electric toothbrush’s vibration is not a significant risk factor for inducing recurrent BPPV.
